# A Custom qPCR Assay to Simultaneously Quantify Human and Microbial DNA

**DOI:** 10.3390/genes15091129

**Published:** 2024-08-27

**Authors:** Miriam Foster, Jennifer A. McElhoe, Mitchell M. Holland

**Affiliations:** Forensic Science Program, Department of Biochemistry & Molecular Biology, Pennsylvania State University, University Park, PA 16802, USA; jam760@psu.edu (J.A.M.); mmh20@psu.edu (M.M.H.)

**Keywords:** DNA quantification, forensic microbiology, skin microbiome, qPCR assay

## Abstract

To date, studies on microbial forensics have focused mainly on sequence analysis and generally do not include information on the quantification of and comparison between the human and bacterial DNA present in forensic samples. Knowing the amount of each type of DNA can be important for determining when and how best to employ bacterial DNA analysis, especially when there is insufficient human DNA for successful short tandem repeat (STR) typing. The goal of this work was to develop a quantitative PCR (qPCR) assay that simultaneously quantifies human and bacterial DNA that would be simple and cost-effective for laboratories to implement. Through a reproducibility study and several small-scale experiments, the reliability of a custom qPCR assay was established. A reproducibility study illustrated that the multiplex assay produced data comparable to that of previously established bacterial DNA and human DNA qPCR assays. The small-scale experiments showed that common surfaces such as keyboards (6.76 pg/μL), elevator buttons (11.9 pg/μL), cleaning supplies (7.17 pg/μL), and dispensers (16.4 pg/μL) failed to produce human DNA quantities sufficient for quality STR analysis (≥250 pg). However, all tested surfaces produced bacterial DNA quantities suitable for reaching 1 ng of amplified bacterial targets necessary for sequence analysis. In fact, bacterial DNA concentrations down to 10^−8^ ng/uL produce enough amplified product for sequencing. The newly developed qPCR multiplex tool will allow scientists to make better decisions regarding whether human or bacterial DNA analysis methods can be pursued during forensic or other investigations.

## 1. Introduction

The field of forensic microbiology allows for additional questions to be considered during the investigation of crimes. The forensic community may have an interest in knowing whether and to what extent microscopic organisms can help determine where something or someone has been, or if there has been contact between two objects. There is also interest in using microbial signatures on a national security level to help assess for threats of bioterrorism [1]. The skin microbiome has the potential to provide significant information for creating associations between people, crime scenes, and geographic areas [2]. It may also help in reconstructing a sequence of events for an investigation. A burgeoning body of work has illustrated how a microbial footprint could be used to identify and individualize humans [3,4,5,6,7]. However, to effectively apply microbial methods, it would be useful to have a better understanding of how much microbial DNA can be recovered from various surfaces and how these results compare to that of human DNA found on the same surface.

One advantage of forensic microbiology is the ability of certain microbes to survive in harsh environments and, therefore, aid in the persistence of their DNA. Certain species of bacteria can protect themselves from radiation, a wide range of temperatures, and low pressures [8]. This resilience to extreme conditions suggests that bacterial DNA is better protected than human DNA. Bacteria have a capsule protecting their DNA from harm that allows them to better stick to surfaces [9]. It is likely that bacterial DNA will persist within a touch sample better and longer than the associated human DNA. It follows that bacterial DNA analysis may then be a viable option for certain touch DNA samples in forensic investigations [10].

There have been numerous studies and reviews on the potential use of microbial DNA in forensic applications [11,12,13,14,15,16,17]. However, there are still gaps in what is known. Many studies that examine the behavior of bacterial DNA in a forensic context fail to compare the amount of bacterial DNA present to that of human DNA [11,18,19]. There is a lack of understanding of how and to what extent bacterial DNA, in comparison to human DNA, can be detected on a surface. Gathering quantitative information will assist in answering the questions of if and when microbial DNA analysis can be used in criminal investigations.

The amount of human DNA in a touch DNA sample is typically variable and present in low quantities making it difficult to analyze [20]. The small amount of human DNA present can be highly susceptible to damage and degradation. Environmental factors such as wind, rain, sunlight exposure, and humidity can carry away or damage DNA at a crime scene, and the small amounts of DNA present in touch samples makes these samples more transient than high DNA concentration samples like blood or saliva [21]. Furthermore, the highly variable amount of DNA that is deposited, along with other factors such as DNA-degrading organisms, makes touch DNA samples vulnerable [22]. Microbial DNA from the skin of an individual will also likely be deposited in a touch DNA sample. One question being investigated by researchers is whether microbial DNA analysis may be better suited for the evaluation of touch DNA when there is limited human DNA present to conduct traditional STR analysis [7].

While there is a growing body of work evaluating the forensic application of microbial methods, information addressing both human and bacterial DNA behavior on forensically relevant surfaces is lacking. Some studies have examined the presence of microbial DNA on surfaces such as mobile phones [18], shoe soles [19], and computer keyboards and mice [11]. For example, in a study by Meadow et al. [18], the association between microbes found on the hands of donors as compared to their personal cell phones was investigated. The authors determined that approximately 22% of the bacterial taxa found on the fingers of the donors were also found on the phones of the donors, as opposed to the 17% of taxa that were found on average to be on the phones of other donors. Lax et al. studied whether an association between shoe soles and the floor on which the participants walked could be determined as well as if the microbial signature on an item, a shoe or phone, could be linked back to the donor [19]. Using supervised learning, the authors found that the shoes and phones could be linked to the individual that owned the item. The authors also concluded that microbial signatures from the shoes were influenced by the geographical area they had walked through and, using a Bayesian method of analysis, that a donor could be linked to a shoe based on the similarity of microbial signatures between the shoe and the floor.

While forensic microbiology is clearly a field of interest within the forensic community, there are still questions to be answered as to how practical it is to use bacterial DNA for forensic investigations. First and foremost, there needs to be an easy and efficient tool to assess the amount of bacterial and human DNA recovered from forensically relevant items. Once armed with such a tool, more information surrounding the expectations of microbial DNA quantities on commonly touched surfaces can be gathered. Furthermore, these data can be compared to that of human DNA to elucidate the benefits of analyzing the different DNA types. Here, we have developed a novel quantitative PCR (qPCR) multiplex to simultaneously quantify human and bacterial DNA. A reproducibility study was conducted to ensure consistent results when compared to the single-plex assays. Amplification of the bacterial DNA amplicon in the form of gBlocks was carried out at concentrations ranging from 10 ng/μL down to 1 × 10^−8^ ng/μL to evaluate whether enough PCR product could be produced for subsequent sequencing. Finally, several surfaces were swabbed to determine how much bacterial and human DNA could be detected on each, and to evaluate what types of surfaces may be better suited for bacterial DNA analysis as opposed to traditional STR analysis. The main objective of this work was to develop a tool to simultaneously quantify human and bacterial sources of DNA and to demonstrate how the tool could serve the forensic science and research communities.

## 2. Materials and Methods

### 2.1. qPCR Assays

Three different qPCR assays were used in this study to assess DNA yields from extracted samples and multiple surfaces; a previously published universal bacterial assay [23], a commercially available human assay [Quantifiler^®^ HP (human plus); ThermoFisher, Waltham, MA, USA], and a custom multiplex qPCR assay combining the bacterial and human targets. A comparison of the parameters for each assay can be found in Table 1. All assays were run on the Applied Biosystems 7500 Real Time qPCR instrument (ThermoFisher, Waltham, MA, USA).

The universal bacterial qPCR assay used primers and a probe targeting the universal 16S rRNA gene as described by Rhitalahti et al. [23] and gBlocks (IDT; San Diego, CA, USA) were used as standards. The gBlocks are chemically synthesized double stranded DNA fragments. While other bacterial DNA qPCR assays have employed bacterial community standards to develop a standard curve [24], this assay uses gBlocks to create a more affordable standard curve template for qPCR [25,26] that is less time-consuming to construct. For the bacterial assay, the synthetic fragment consisted of a 366-base pair (q) stretch of DNA corresponding to the bacterial target amplicon. The gBlock stock (10 ng/μL) was serially diluted by a factor of 10X from 1 ng/μL to 10^−6^ ng/μL. The universal bacterial reaction consisted of 5.4 μL 10X buffer containing Mg^2+^ (Takara Bio USA, San Jose, CA, USA), 2.4 μL dNTPs (2.5 mM each; Takara Bio USA, San Jose, CA, USA), 0.39 μL bovine serum albumin (BSA, 10 mg/μL; Invitrogen, Carlsbad, CA, USA), 0.25 μL Hot Start Taq (5 U/μL; Takara Bio USA, San Jose, CA, USA), 2 μL forward primer (4.5 μM; IDT, San Diego, CA, USA), 2 μL reverse primer (4.5 μM; IDT, San Diego, CA, USA), 2 μL probe (4.5 μM with FAM dye and a MGB quencher; ThermoFisher, Waltham, MA, USA), 13.56 μL PCR grade water (Promega, Madison, WI, USA), and 2 μL of standards or DNA template, for a total reaction volume of 30 μL. The cycling parameters were as follows: an initial soak at 50 °C for 2 min and at 95°C for 10 min, followed by 40 cycles of PCR at 95 °C for 15 s, 52 °C for 1 min, and 72 °C for 1 min.

For the human DNA components of this study the Quantifiler^®^ HP kit (ThermoFisher, Waltham, MA, USA) was used. This system targets a small, multilocus autosomal region of the human genome (80 bp) for quantification purposes, as well as a large, multilocus autosomal target (214 bp) to facilitate the assessment of degradation [27]. The HP kit also allows for the evaluation of inhibition through a synthetic internal PCR control (IPC). The kit includes THP DNA Standard (100 ng/μL) and THP DNA Dilution Buffer which are used to create five, serially diluted human DNA standards at concentrations of 50, 5, 0.5, 0.05, and 0.005 ng/μL. When run solely as a human DNA qPCR assay, 10 μL of the Reaction Mix and 8 μL of the Primer Mix were combined for each reaction followed by 2 μL of standards or DNA template to reach a total volume of 20 μL. The cycling parameters were those prescribed by the manufacturer.

The custom multiplex, including the human and bacterial targets, consisted of the Quantifiler^®^ HP kit and the bacterial primers and probe from the custom universal bacterial assay. Each qPCR reaction consisted of 10 μL HP Primer Mix, 12.5 μL HP Reaction Mix, 0.15 μL bacterial forward primer (50 μM), 0.15 μL bacterial reverse primer (50 μM), 0.15 μL bacterial probe (50 μM), 0.05 μL PCR grade water, and 2 μL of standard or DNA template for a total reaction volume of 25 μL. The cycling parameters were the same for those of the Quantifiler^®^ HP assay. The 7500-instrument HID software allows for the analysis of three dyes in custom mode. As this study centered around quantification and potential inhibition, the small autosomal target was used for reporting human quantities and the IPC was used for assessment of observed inhibition; with the bacterial target as the third reporter dye. Future studies may include an evaluation of degradation by replacing the IPC for the large target dye.

### 2.2. Reproducibility Study

To evaluate the consistency of results between the single-plex assays and the multiplex, as well as within each assay, a reproducibility study was conducted. A sample with known human DNA amounts was obtained by extracting DNA from a whole blood sample using the Zymo Quick-DNA Miniprep Plus Kit (Zymo Research; Irvine, CA, USA) according to the manufacturer’s protocol. This extract was then quantified using the Quantifiler HP kit and was found to have a human DNA concentration of 26 ng/μL. This sample was then diluted to concentrations of 13, 6.5, 1, and 0.25 ng/μL.

The original DNA extract and the dilutions were then quantified with the HP kit, alongside two sets of the HP standards, to obtain data for the first round of the reproducibility study. The bacterial assay was run with two sets of the gBlock dilutions as standards (1, 0.1, 0.01, 1 × 10^−3^, 1 × 10^−4^, 1 × 10^−5^, and 1 × 10^−6^ ng/μL) and a third set run as unknown samples (1, 0.1, 0.01, 1 × 10^−3^, 1 × 10^−4^, 1 × 10^−5^, and 1 × 10^−6^ ng/μL). Finally, the multiplex was run with two sets of the HP standards (50, 5, 0.5, 0.05, and 0.005 ng/μL), two sets of the gBlocks bacterial standards (1, 0.1, 0.01, 1 × 10^−3^, 1 × 10^−4^, 1 × 10^−5^, and 1 × 10^−6^ ng/μL), one set of the human known samples (26, 13, 6.5, 1, and 0.25 ng/μL), and another set of the gBlocks as the bacterial known samples (1, 0.1, 0.01, 1 × 10^−3^, 1 × 10^−4^, 1 × 10^−5^, and 1 × 10^−6^ ng/μL). This experiment was repeated five times.

### 2.3. Bacterial DNA Amplification

An input of 1 ng DNA is typically required for bacterial DNA sequencing [3]. To determine if initial bacterial DNA quantities being detected on surfaces could be amplified to reach the 1 ng threshold for sequencing and further analysis, an experiment was performed in which gBlocks were amplified using PCR. A TapeStation (Agilent Technologies, Santa Clara, CA, USA) was used to quantify the PCR products generated using various input concentrations of the gBlock standard. The upper end of the input DNA range was 10 ng/μL which then underwent a 10X serial dilution down to 1 × 10^−8^ ng/μL. As 2 μL of each dilution was used, the total input template range was 20 ng to 2 × 10^−8^ ng. The serially diluted gBlocks were amplified in duplicate. The general PCR recipe suggested by Takara [28] was modified for a 25 μL reaction. Each PCR reaction consisted of 2.5 uL 10X buffer with Mg^2+^ (Takara Bio USA, San Jose, CA, USA), 2 μL dNTP mix (2.5 mM each; Takara Bio USA, San Jose, CA, USA), 0.125 μL of Hot Start Taq (5 U/μL; Takara Bio USA, San Jose, CA, USA), 0.15 μL each universal bacteria primers (50 μM; IDT, San Diego, CA, USA), 18.075 μL PCR grade water (Promega, Madison, WI), and 2 μL of gBlock template, for a total reaction volume of 25 μL. The cycling parameters used were those suggested by Takara: soak at 94 °C for 2 min, followed by 30 cycles of 98 °C for 10 s, 55 °C for 30 s, and 72 °C for 1 min. The amplified products were quantified by Penn State University’s Genomics Core Facility in the Huck Institute on a TapeStation D5000 ScreenTape instrument (Agilent, Santa Clara, CA, USA).

### 2.4. Dataset Creation

A total of eleven surfaces were tested in this study (Table 2). The purpose of this study was to illustrate the value of the multiplex assay, so the conditions under which each surface was touched, and the cleaning regime for each surface type, were not controlled. Each individual surface was swabbed once for 30 s with a UV-irradiated small, tapered cotton swab (Puritan Medical Products, Guilford, ME). The swabs were moistened with 20 μL of 10 mM TRIS-HCL, 0.1% TWEEN-20, pH 8.5 (Teknova, Hollister, CA, USA) and excess liquid was removed prior to swabbing.

### 2.5. DNA Extraction of Surface Swabs

DNA was isolated using the ZymoBIOMICS DNA Miniprep Kit (Zymo Research, Irvine, CA, USA) according to the manufacturer’s protocol except for the addition of Lysozyme. When adding the 750 μL of lysis buffer to the swab, 15.0 mg of powdered lyophilized Lysozyme (ThermoFisher, Waltham, MA, USA) was added for a final concentration of 20 mg/mL. The tube was then incubated at 37 °C for 30 min [29]. After incubation, the tubes were vortexed on the max setting of a Vortex Genie 2 (Scientific Industries, Inc., Bohemia, NY, USA) for 40 min. To increase yields, the manufacturer’s suggested low DNA yield elution procedure was followed. The RNase/DNase Free Water used for elution of the DNA was heated to 60 °C prior to elution in a final volume of 50 μL. The eluate was then run through the column matrix a second time. The eluate was stored in a 4 °C fridge (short-term) or in a −20 °C freezer (long-term). For each extraction round, a blank, moistened, swab was processed to assess potential contamination. Extracts were then quantified using the custom multiplex qPCR assay.

### 2.6. Data Analysis

Statistical analysis was completed using R [30] and R Studio [31]. Multiple comparison testing was performed after testing for normality with the Shapiro–Wilk test (R stats package v.4.3.1) [32]. Non-parametric multiple comparison testing was used for the non-normally distributed datasets using the Kruskal–Wallis rank test (R stats package v.4.3.1) [33] and Dunn’s post hoc testing [34,35] with Holm correction. Ggplot2 (v.3.4.2) [36] was used for figure generation. Bland–Altman plots comparing the reproducibility study results were also created in R [37,38].

## 3. Results and Discussion

### 3.1. Multiplex qPCR Assay Development and Reproducibility Study

**Figure 1:**
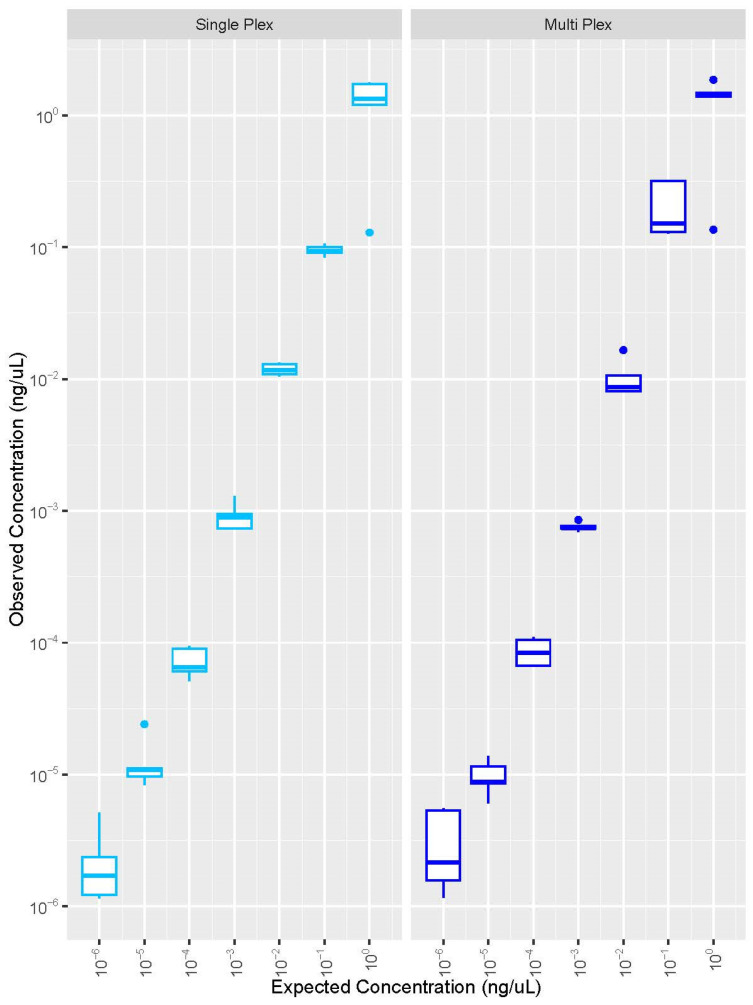
Expected and observed concentrations for the universal bacterial DNA reproducibility study (n = 5). The left panel presents the data from the custom bacterial single-plex, while the right panel represents the data for the bacterial component of the multiplex assay. Note: A log10 scale was used for the *y*-axis. The middle bar represents the median.

Through Shapiro–Wilk testing, the data associated with the bacterial DNA study was found to be non-normally distributed (*p*-value = 1.315 × 10^−14^). The bacterial DNA quantification data were similar when comparing the single-plex and multiplex assays (Figure 1). The Kruskal–Wallis multiple comparison testing indicated no significant difference between any of the comparisons (*p* > 0.05; Table S1).

**Figure 2:**
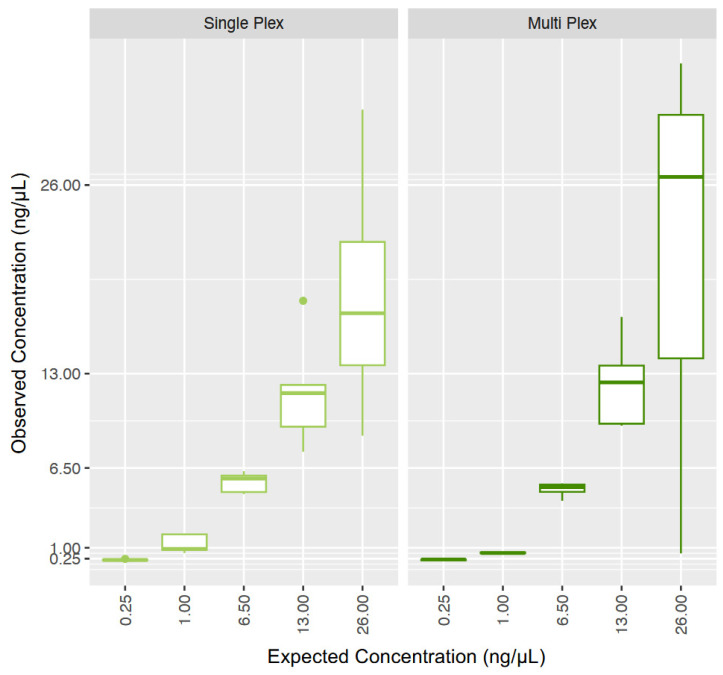
Expected and observed concentrations for the human DNA reproducibility study (n = 5). The left panel presents the data from the Quantifiler^®^ HP assay alone, while the right panel represents the data for the human component of the multiplex assay. The middle bar represents the median.

Through Shapiro–Wilk testing, the data associated with the human DNA study were found to be non-normally distributed (*p*-value = 1.154 × 10^−6^). The human DNA quantification data was similar when comparing the HP and multiplex assays (Figure 2), with greater variability at the highest quantity, consistent with expectations. The Kruskal–Wallis multiple comparison testing indicated no significant difference between any of the comparisons (*p* > 0.05; Table S2).

**Figure 3:**
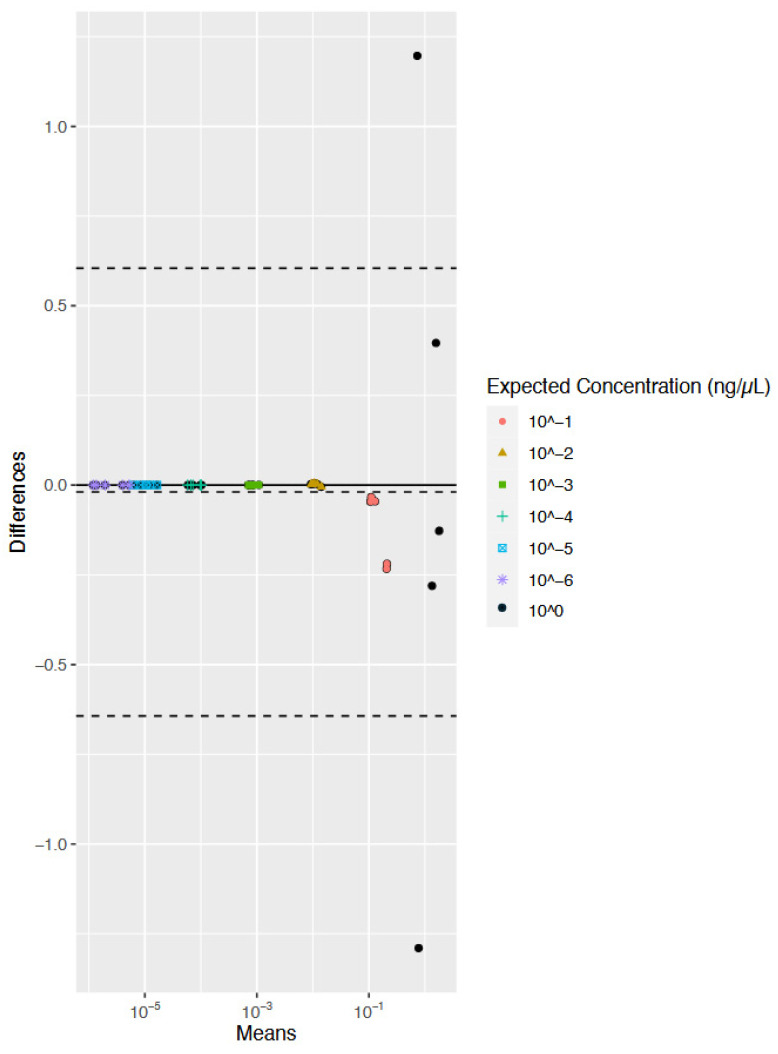
Bland–Altman plot comparing the bacterial single-plex data to the bacterial DNA component of the multiplex. The X-axis represents the mean of one observation as measured by the bacterial single-plex assay and the multiplex for each replicate (n = 5) for each expected concentration (n = 7). The Y-axis represents the difference of the same two observations for each replicate for each expected concentration. The dashed lines represent the mean of differences (middle line = bias) and the limits of agreement mean of +/−1.96 standard deviations (S.D.) (upper and lower lines, respectively). Note: Bland–Altman plots do not have units for the X- and Y-axis. The key reflects values of, for example, 10^−1 as 10^−1^, with the last entry (10^0) as the most concentrated samples.

Even though the sample size for the reproducibility study was small (n = 5 for each expected concentration), a preliminary statistical evaluation of the agreement between the single-plex and multiplex was conducted for both the human and the bacterial qPCR assays. Bland–Altman plots show the comparison of two methods of measurement to determine if the methods are interchangeable [39]. More specifically, the Bland–Altman method [38] was used to assess and visualize the degree of agreement between each of the two measurement methods and identify any systematic bias. Data are plotted on the X- and Y-axis, with X representing the mean of the two measurements and Y representing the difference between the two measurements. For example, for the bacterial data (Figure 3), an observation of the sample expected to have a concentration of 1 ng/μL as measured by the bacterial single-plex assay was compared to an observation of the same sample quantified with the multiplex assay. The difference and mean of these data points were calculated and plotted on the graph. The range of the limits of agreement (LoA) is an indication of interchangeability of the two assays. The LoA dictates the bounds of the data based on +/−1.96 S.D. from the average of the data. Bland and Altman suggest that 95% of the data should fall between these bounds [38]. Interpretation of the graph requires the determination of acceptable a priori considerations.

The Bland–Altman plot for the bacterial data shows a bias of −0.02, as represented by the center-most dashed line, demonstrating the bacterial component of the multiplex measures 0.02 units more than the bacterial single-plex, on average (Figure 3). As the data were more scattered towards the right of the graph, it suggests the variability of the data increases as the means increase. This scattering drives the average bias towards −0.02. This level of bias reflects that more variability is observed in samples with higher quantification values. While the bias can be a constant, for this dataset the overall underestimation is most likely a result of differences in the higher concentration values (0.1 and 1 ng/μL) and is reflected in the figure with the difference increasing as the averages increase. The upper limit of agreement (ULoA) is 0.60, while the lower limit of agreement (LLoA) is −0.64 (Figure 3). Given that only two out of 35 total data points are outside of the range (~5.7%), and that the quantification dynamic ranges are so large, overall, the bacterial single-plex method and the custom multiplex method produce similar quantification values. Furthermore, the range of the LoAs for the bacterial data is relatively narrow, and in conjunction with the small bias, suggests the assays are approximately equivalent. It is important to note that this assessment would have more statistical power with larger sample sizes.

**Figure 4:**
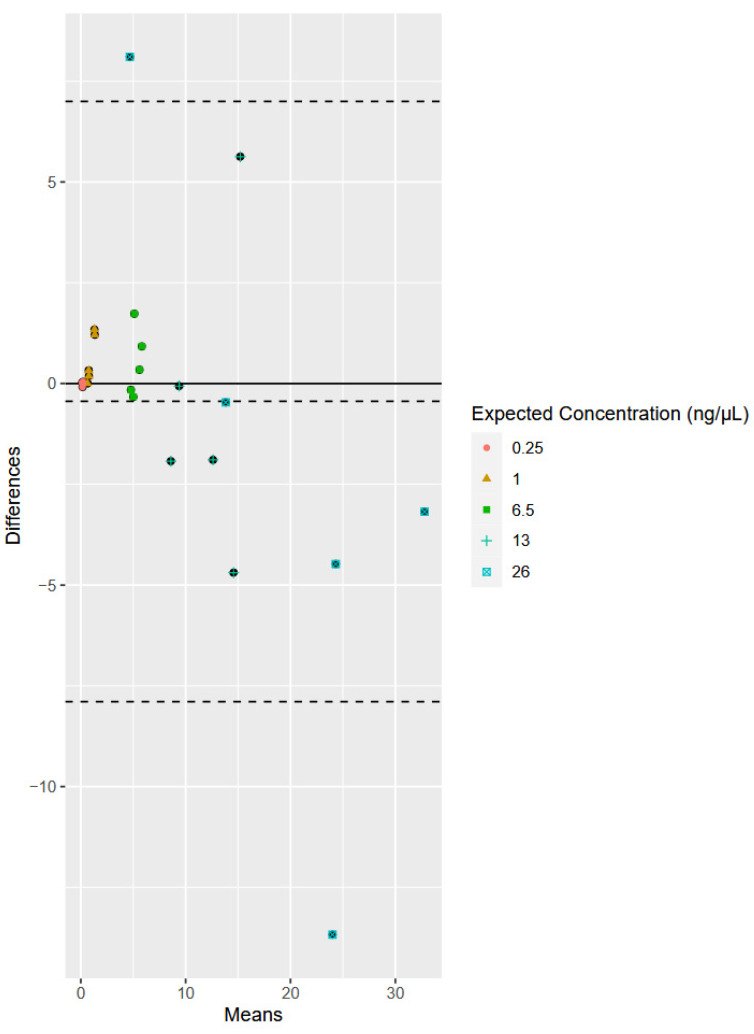
Bland–Altman plots comparing the HP data to the human component of the multiplex. The X-axis represents the mean of one observation as measured by the HP single-plex assay and the multiplex for each replicate (n = 5) for each expected concentration (n = 5). The Y-axis represents the difference of the same two observations for each replicate for each expected concentration. The dashed lines represent the mean of differences (bias) and the limits of agreement (LoAs) mean +/− 1.96 S.D. Note: Bland–Altman plots do not have units for the X- and Y-axis.

The Bland–Altman plot for the human data shows a bias of −0.45, which shows that, on average, the human component of the multiplex measures 0.45 units more than the HP kit (Figure 4). Once again, the overall underestimation is most likely a result of differences in the higher concentration values (13 and 26 ng/μL) and is reflected in the figure with the difference increasing as the averages increase. While this level of bias is higher than that seen for the bacterial system, it is consistent with the majority of the variability being found in the higher quantification values, and the dynamic range of quantification values. The ULoA is 7.00, while the LLoA is −7.89 (Figure 4). There are 2 of 25 data points that fall outside of the range (8%). Nonetheless, overall, the human single-plex and custom multiplex methods produce similar quantification values, especially for samples with concentrations below 26 ng/μL. As is typically employed, a sample found to have a concentration at the higher end of the dynamic range of a qPCR assay can be diluted and quantified again for a more accurate measurement of quantification.

### 3.2. Bacterial DNA Amplification

**Figure 5:**
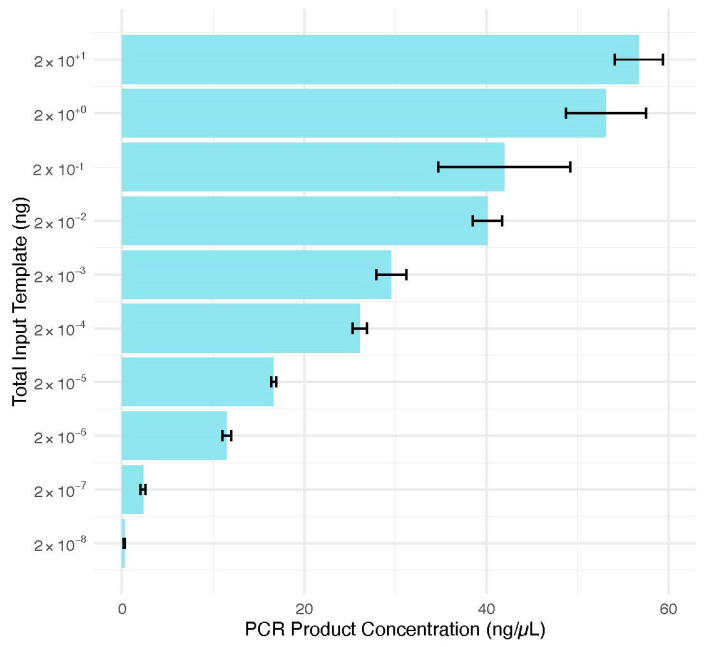
Average quantification results for bacterial DNA amplifications. Synthetic bacterial DNA was amplified in duplicate (n = 2) with a total template input range of 20 to 2 × 10^−8^ ng. Error bars represent the mean +/− standard error.

The average PCR product concentrations (ng/μL) for the 20 (56.8 ± 3.7), 2 (53.1 ± 6.2), 0.2 (42.0 ± 10.3), 2 × 10^−2^ (40.1 ± 2.3), 2 × 10^−3^ (29.6 ± 2.3), 2 × 10^−4^ (26.1 ± 1.1), 2 × 10^−5^ (16.7 ± 0.3), 2 × 10^−6^ (11.5 ± 0.7), and 2 × 10^−7^ (2.31 ± 0.4) ng input templates surpassed the necessary amount to allow for a 1 ng input for subsequent bacterial DNA sequencing (Figure 5). The 2 × 10^−8^ ng input template amount produced an average of 0.251 ± 0.1 ng/μL of PCR product. According to the manufacturer’s protocol for the Nextera XT DNA Library Preparation Kit (Illumina; San Diego, CA, USA), 5 μL of DNA can be used [40]. Therefore, concentrations of bacterial DNA of at least 1 × 10^−8^ ng/μL will be sufficient for bacterial DNA analysis. These quantification results demonstrated that bacterial DNA quantities down to 10^−8^ ng/μL can successfully generate PCR products in quantities sufficient for subsequent sequence analysis. Further work should be performed to evaluate the limit of sensitivity for the amplification of bacterial DNA below template input amounts of 2 × 10^−8^ ng. For samples lacking enough bacterial DNA, a nested PCR approach could be attempted to produce PCR product sufficient for sequence analysis. In addition, this assumes that a visible PCR product on a TapeStation result indicates the potential for a sample to be sequenced. Knowing the range of input bacterial DNA that can be amplified and consequently sequenced will allow scientists to make better decisions regarding whether human or bacterial DNA analysis methods can be pursued during forensic or other investigations.

### 3.3. Dataset Creation

Most sample types in this study produced low levels of both human and bacterial DNA. While, on average, the amount of human DNA was greater than that of the bacterial DNA in every sample, only some of the sample types reached an average concentration that allows for the addition of at least 250 pgs of template DNA to an STR reaction. This template amount is desired for obtaining quality STR profiles [41]. The sample types with high enough concentrations were the computer mouse, smart watch, sebum deposit, fridge handle, dresser drawer, light switch, and TV remote, which had average concentrations of approximately 76, 357, 59, 39, 56, 97, and 165 pg/μL, respectively (Table 3). According to the PowerPlex^®^ Fusion 6C (Promega; Madison, WI, USA) technical manual, a maximum volume of 15 μL of sample can be added to a 25 μL STR amplification reaction, allowing these samples to surpass the 250 pg human DNA threshold [42].

It should be noted that while the computer mouse surface type reached the STR threshold on average, only two out of eight individual samples reached the threshold for human DNA analysis. In total, 8 out of 10 smart watch samples, 6 out of 10 sebum deposit samples, 1 out of 2 fridge handle samples, both dresser drawer samples, all 3 light switch samples, and both TV remote samples reached the threshold for human DNA analysis. The remaining sample types, on average, fell short of 250 pg of human DNA, even with the maximum volume of sample added, suggesting the remaining sample types would be less successful when undergoing STR analysis. More specifically, none of the eight keyboard samples, one out of four elevator button samples, none of the cleaning supplies samples, and one out of four dispenser samples reached the threshold for human STR analysis.

All sample types, on average, produced high enough concentrations, 1 × 10^−8^ ng/μL or greater, for bacterial DNA sequence analysis. Furthermore, all individual samples met the threshold for bacterial DNA analysis.

When analyzing data from the multiplex runs, higher than expected IPC values were observed for surface samples (Table 3). To determine whether this indicated inhibition, samples spiked with known amounts of DNA were quantified. The expected amounts of DNA detected in these samples suggest that the high IPC values did not indicate inhibition. Therefore, when using the multiplex assay, it will be important to adjust the threshold for inhibition observed in forensic samples.

Based on the compiled quantification data for different surface sources, some preliminary information can be gathered. High traffic, rarely cleaned surfaces, such as keyboards and computer mice in a computer lab, did not contain as much human or bacterial DNA as expected. This was also observed for soap and hand sanitizer dispenser and elevator button samples, which were cleaned daily. One possible reason for the lack of DNA could be that as people touch items, they not only deposit DNA, but also remove DNA from the surface. In addition, some surfaces may not be as receptive for retaining biological material.

A study by Fierer et al. [11] sampled computer keys and mice, however, they focused on examining if microbial footprints could differentiate between the individuals who handled these objects. The authors found that the microbial signatures from the finger of an individual were unique to that individual and the differences in microbial communities between individuals were greater than any differences observed on the fingers and keyboards belonging to a single individual. Fierer et al. [11] also found that a skin swab of an individual stored at 20 °C for two weeks maintained its microbial composition and could still be used to differentiate between individuals. The study did not, however, provide quantification results for their microbial DNA data, nor did they include the comparison to human data. The work performed in this study allows for that gap to be bridged and to bring more context to studies such as these.

In a study by Schmedes et al. [3], the authors developed a tool to identify specific skin microbiome taxa that can be used to potentially profile individuals. Though this study garnered positive results, it focused on the swabbing of hands, rather than the swabbing of surfaces after the deposition of DNA. The results most likely will be different when transfer is involved as less DNA will be available compared to the amounts of DNA found directly on skin. A gap left by the Schmedes et al. study [3] was the lack of quantitative information. Instead, the study centered around characterizing skin microbiome profiles. The authors mentioned the use of the Qubit Fluorometer 2.0 to quantify samples at different points in the study, but the data were not reported, and this is a non-specific method of quantification. The authors stated that for some samples, the amount of human DNA taken from a donor fell below the ideal input requirement of 1 ng for the Illumina ForenSeq™ panel on the MiSeq FGX Forensic Genomics System (Illumina; San Diego, CA, USA), but they did not delve into the comparison between microbial and human DNA quantities [3]. Using the novel qPCR assay developed in this work, the quantification of microbial and human DNA could be assessed for the hands of donors and compared to the quantities found on surfaces. The furthering of this work will allow for a more in-depth understanding of when and why bacterial DNA analysis may benefit the field of forensics.

Lastly, Seashols-Williams et al. [24] examined the quantities of human and bacterial DNA in different human body fluids. They found that much higher average amounts of human (2.2 to 6134.9 ng) and bacterial (0.3 to 322.1 ng) DNA were detected within body fluids, as compared to our results from surfaces. The authors did find that in some cases, there was a higher ratio of bacterial to human DNA. While the results of our study did not demonstrate instances of higher amounts of bacterial DNA compared to human DNA, they did demonstrate that, in some cases, bacterial DNA analysis may be more appropriate than human DNA analysis. The Seashols-Williams et al. study [24] provides insight into the quantities of human and microbial DNA in body fluids, but once again, does not link the two assays for convenience and efficiency. We recommend that future studies include a quantification assessment of samples removed from surfaces, preferably ones that include both bacterial and human DNA targets.

## 4. Conclusions

The majority of current research in forensic microbiology centers around sequence analysis of microbial DNA, without providing information on the quantity of bacterial DNA present, or without comparing it to the amount of recovered human DNA. This work aimed to provide a tool to bridge that gap. The novel qPCR multiplex provides reliable quantification information for human and bacterial DNA in a single assay. The incorporation of the commercially available Quantifiler^®^ HP kit made the use of this assay simple and cost-effective. As many forensic laboratories already use the Quantifiler^®^ HP system, they could easily transition to the multiplex assay. It is simple to use, is cost effective, and is reliable.

Future work should evaluate the persistence of both types of DNA on a variety of surfaces. If it is found that human DNA degrades quicker than bacterial DNA, there would be more cause to pursue bacterial DNA methods. Additionally, the effects of microbial community growth and change compared to the stagnant or decreasing nature of human DNA will need to be considered. Lastly, this is a proof-of-concept study for bacterial DNA. Other qPCR assays could be developed for fungal and other sources of non-human DNA.

## Figures and Tables

**Table 1:** Comparison of assay parameters for both single-plex and the multiplex assays. The number of targets, master mix components, final volumes, and cycling parameters are listed. The multiplex was comprised of a mix of the single-plex assay conditions.

Assay	Quantifiler^®^ HP	Bacterial Single-Plex	Multiplex
Number of Targets	Two	One	Three
Master Mix components	HP Reaction and Primer Mixes	10× buffer containing Mg^2+^, dNTPs, BSA, Hot Start Taq, bacterial primers and probe, PCR grade water	HP Reaction and Primer Mixes, bacterial primers and probe, PCR grade water
Final volume	20 μL	30 μL	25 μL
Cycling parameters	Holding stage at 95 °C for 2 min, 40 cycles of 95 °C for 9 s, 60 °C for 30 s	Initial stage at 50 °C for 2 min, 95 °C for 10 min, 40 cycles of 95 °C for 15 s, 52 °C for 1 min, 72 °C for 1 min	Holding stage at 95 °C for 2 min, 40 cycles of 95 °C for 9 s, 60 °C for 30 s

**Table 2:** Description of sampling areas for each surface type, as well as the number of samples for each surface type. Computer components were in a computer laboratory. Smart watch and sebum deposit samples were sourced from volunteers. Elevator buttons, cleaning supplies, and dispensers were in high-traffic areas. Fridge handles, dresser drawers, light switches, and TV remotes were in the apartments of three volunteers. The N value represents the number of samples for each surface type.

Sample Type	N	Description
Keyboard	8	Enter, shift, “e,” “r,” “t,” “a,” “s,” “d,” “f,” “g,” “h,” “j,” “k,” “l,” ctrl, and fn keys
Computer Mouse	8	Sides, top, and roller
Smart Watch	10	Entire band, back of screen
Elevator button	4	Buttons and immediate outside area
Cleaning supplies	4	Upper 1/3 of handle
Sebum deposit	10	Forehead of donor was rubbed; fingertip was placed on desktop
Dispenser	4	Pump of each one hand sanitizer dispenser and three hand soap dispensers
Fridge Handle	2	Entirety of handle of fridge
Dresser Drawer	2	Entirety of handle for each drawer of dresser
Light Switch	3	Entirety of switch, not panel
TV Remote	2	Front, back, side, and in between buttons

**Table 3:** Human and bacterial DNA concentrations (average and standard deviation) for each surface type, along with average internal PCR control (IPC) value (C_T_ or cycle threshold values). The N value represents the number of samples for each surface type.

Sample Type	N	Average Human DNA Quantity (ng/μL)	Standard Deviation	Avg Bacterial DNA Quantity (ng/μL)	Standard Deviation	Average IPC (C_T_ Value)
Keyboard	8	0.0075	0.006	2.21 × 10^−7^	1.40 × 10^−7^	29.7
Mouse	8	0.076	0.183	2.52 × 10^−7^	1.42 × 10^−7^	29.5
Smart Watch	10	0.357	0.426	1.14 × 10^−5^	1.28 × 10^−5^	29.7
Elevator button	4	0.012	0.011	2.13 × 10^−7^	1.39 × 10^−7^	29.7
Cleaning supplies	4	0.007	0.005	4.40 × 10^−7^	5.15 × 10^−7^	29.6
Sebum deposit	10	0.059	0.060	4.76 × 10^−7^	5.23 × 10^−7^	29.8
Dispensers	4	0.016	0.013	4.71 × 10^−7^	3.43 × 10^−7^	29.4
Fridge Handle	2	0.039	0.049	8.34 × 10^−8^	9.52 × 10^−8^	29.9
Dresser Drawer	2	0.056	0.020	2.45 × 10^−7^	1.22 × 10^−7^	29.8
Light Switch	3	0.097	0.057	2.83 × 10^−7^	1.79 × 10^−7^	29.8
TV Remote	2	0.165	0.040	3.98 × 10^−7^	4.63 × 10^−7^	29.7

## Data Availability

The raw data supporting the conclusions of this article will be made available by the authors on request.

## Supplementary Materials

The following supporting information can be downloaded at: https://www.mdpi.com/article/10.3390/genes15091129/s1, Table S1: Statistical Comparison Between Universal Bacterial Single-plex and Bacterial DNA Component of Multiplex and Table S2: Statistical Comparison Between Quantifiler^®^ HP Kit and Human DNA Component of Multiplex.
