# Supplementary material for: A Custom qPCR Assay to Simultaneously Quantify Human and Microbial DNA
Source: Genes (Basel). 2024 Aug 27;15(9):1129. doi: 10.3390/genes15091129 (PMC11431276; doi:10.3390/genes15091129)
Supplement: Supplementary file 1 [file genes-15-01129-s001.zip › Table S1.pdf]

Table S1: Statistical Comparison Between Universal Bacterial Single-plex and Bacterial DNA Component of Multiplex. The Dunn post-hoc test with the Holm adjustment method was conducted to evaluate any significant difference between the bacterial single-plex data and the data from the human component of the multiplex.

Comparison	P value
Bacterial singleplex observations vs bacterial component of the multiplex observations for the 1 ng/uL expected value	0.950
Bacterial singleplex observations vs bacterial component of the multiplex observations for the 0.1 ng/uL expected value	1.00
Bacterial singleplex observations vs bacterial component of the multiplex observations for the 0.01 ng/uL expected value	1.00
Bacterial singleplex observations vs bacterial component of the multiplex observations for the 1 x 10^{-3} ng/uL expected value	1.00
Bacterial singleplex observations vs bacterial component of the multiplex observations for the 1 x 10^{-4} ng/uL expected value	1.00
Bacterial singleplex observations vs bacterial component of the multiplex observations for the 1 x 10^{-5} ng/uL expected value	1.00
Bacterial singleplex observations vs bacterial component of the multiplex observations for the 1 x 10^{-6} ng/uL expected value	1.00
