# Supplementary material for: A Custom qPCR Assay to Simultaneously Quantify Human and Microbial DNA
Source: Genes (Basel). 2024 Aug 27;15(9):1129. doi: 10.3390/genes15091129 (PMC11431276; doi:10.3390/genes15091129)
Supplement: Supplementary file 1 [file genes-15-01129-s001.zip › Table S2.pdf]

Table S2: Statistical Comparison Between Quantifiler® HP Kit and Human DNA Component of Multiplex.
The Dunn post-hoc test with the Holm adjustment method was conducted to evaluate any significant difference between the Quantifiler® HP Kit data and the data from the human component of the multiplex.

| Comparison | P Value |
|---|---|
| HP observations vs human component of the multiplex observations for the 26 ng/uL expected value | 1.00 |
| HP observations vs human component of the multiplex observations for the 13 ng/uL expected value | 1.00 |
| HP observations vs human component of the multiplex observations for the 6.5 ng/uL expected value | 1.00 |
| HP observations vs human component of the multiplex observations for the 1 ng/uL expected value | 0.896 |
| HP observations vs human component of the multiplex observations for the 0.25 ng/uL expected value | 1.00 |
