# Supplementary material for: A Custom qPCR Assay to Simultaneously Quantify Human and Microbial DNA
Source: Genes (Basel). 2024 Aug 27;15(9):1129. doi: 10.3390/genes15091129 (PMC11431276; doi:10.3390/genes15091129)
Supplement: Supplementary file 1 [file genes-15-01129-s001.zip › Table S3.pdf]

Table S3: Human and bacterial DNA concentrations (average and standard deviation) for each surface type. N represents the number of samples for each surface type.

**Sample Type**	**N**	**Avg human DNA (ng/uL)**	**std dev**	**Avg bacterial DNA (ng/uL)**	**std dev**
Keyboard	8	0.0075	0.006	2.21E-07	1.40E-07
Mouse	8	0.076	0.183	2.52E-07	1.42E-07
Smart Watch	10	0.357	0.426	1.14E-05	1.28E-05
Elevator button	4	0.012	0.011	2.13E-07	1.39E-07
Cleaning supplies	4	0.007	0.005	4.40E-07	5.15E-07
Sebum deposit	10	0.059	0.060	4.76E-07	5.23E-07
Dispensers	4	0.016	0.013	4.71E-07	3.43E-07
Fridge Handle	2	0.039	0.049	8.34E-08	9.52E-08
Dresser Drawer	2	0.056	0.020	2.45E-07	1.22E-07
Light Switch	3	0.097	0.057	2.83E-07	1.79E-07
TV Remote	2	0.165	0.040	3.98E-07	4.63E-07
